# *LEPR* gene Gln223Arg polymorphism and type 2 diabetes mellitus: a meta-analysis of 3,367 subjects

**DOI:** 10.18632/oncotarget.18720

**Published:** 2017-06-27

**Authors:** Yan-Yan Li, Hui Wang, Xin-Xing Yang, Jing-Jing Wu, Hong-Yu Geng, Hyun Jun Kim, Zhi-Jian Yang, Lian-Sheng Wang

**Affiliations:** ^1^ Department of Gerontology, First Affiliated Hospital of Nanjing Medical University, Nanjing 210029, China; ^2^ Department of Cardiology, First Affiliated Hospital of Nanjing Medical University, Nanjing 210029, China; ^3^ Department of Nephrology, First Affiliated Hospital of Nanjing Medical University, Nanjing 210029, China; ^4^ Department of Physiology, University of Cincinnati, Cincinnati, OH 45267, United States of America

**Keywords:** *leptin receptor*, Gln223Arg, polymorphism, type 2 diabetes mellitus

## Abstract

**Background:**

The *Leptin receptor* (*LEPR*) Gln223Arg gene polymorphism has been associated with an increased susceptibility to Type 2 diabetes mellitus (T2DM). Results from various studies, however, are inconsistent.

**Objective and methods:**

To better elucidate the association of *LEPR* Gln223Arg gene polymorphism with T2DM in the Chinese population, a meta-analysis of 3,367 subjects from seven independent studies was performed. The pooled odds ratios (ORs) and corresponding 95% confidence intervals (95% CI) were evaluated by the fixed-effects model.

**Results:**

A significant relationship between *LEPR* Gln223Arg gene polymorphism and T2DM in the Chinese population was found under allele (OR: 1.432, 95% CI: 1.211-1.694, P=2.67×10^-5^), dominant (OR: 1.466, 95% CI: 1.215-1.769, P=6.33×10^-5^), recessive (OR: 0.539, 95% CI: 0.303-0.960, P=0.036), heterozygous (OR: 0.700, 95% CI: 0.577-0.849, P=3.06×10^-4^), and homozygous (OR: 0.472, 95% CI: 0.265-0.839, P=0.011) genetic models.

**Conclusions:**

*LEPR* Gln223Arg gene polymorphism was associated with an increased risk of T2DM in the Chinese population. Therefore, Chinese carriers of the G allele of *LEPR* Gln223Arg gene polymorphism may be more susceptible to T2DM than the general population.

## INTRODUCTION

The most recent survey from the Chinese Diabetes Society shows that the overall prevalence of diabetes mellitus (DM) has increased to 11.6% in the Chinese adult population, making the number of DM patients in China 114 million. This suggests that China, as a country, has the most DM patients in the world [1]. Type 2 diabetes mellitus (T2DM) is a chronic disease associated with abnormal glucose metabolism due to insulin resistance. The pathogenesis of the disease is complicated due to the dynamic interplay between both genetic and environmental factors.

Leptin is peptide hormone secreted by adipose tissue and while it is most famous as the mediator for the sensation of satiety, it also helps regulate energy metabolism and body lipid homeostasis. It also regulates insulin secretion through two distinct mechanisms. It concurrently inhibits parasympathetic nervous system and excites the sympathetic by inhibiting the expression of *neuropeptide Y (NPY)* gene product, ultimately reducing insulin secretion. Leptin also binds with leptin receptor (LEPR) located on pancreatic beta cells to regulate insulin secretion [2, 3].

The *LEPR* gene, located on the chromosome 1p31, spans more than 70 kb, contains 20 exons and 19 introns, and encodes for 1165 amino acids. Though there are many mutations and polymorphisms in the human *LEPR* gene, the polymorphisms sites have been limited almost exclusively to the 20 exons of the *LEPR* gene*.* The Gln223Arg gene polymorphism specifically is caused by the substitution of the adenine at the 668^th^ position by a guanine, mutating the 223^rd^ amino acid, an arginine, to be replaced by a glutamine. This mutation is located on the 6^th^ exon of the protein and may impair signal transduction enough to increase susceptibility to T2DM [4].

Although many studies on the relationship between the *LEPR* Gln223Arg gene polymorphism and T2DM have been conducted in the Chinese population, results are contradictory. In 2009, Ying et al. reported the A allele of *LEPR* Gln223Arg gene polymorphism to be associated with an increased risk of T2DM [5]. In contrast, Fang et al found that the G allele of *LEPR* Gln223Arg gene polymorphism to be a risk factor for the T2DM in 2011[6]. Shi et al obtained the similar conclusion in 2005 [7].

In the current meta-analysis, data from 2,084 T2DM patients and 1,283 controls was analyzed to further elucidate the relationship between *LEPR* Gln223Arg gene polymorphism and T2DM in the Chinese population.

## RESULTS

### Studies and populations

**Figure 1:**
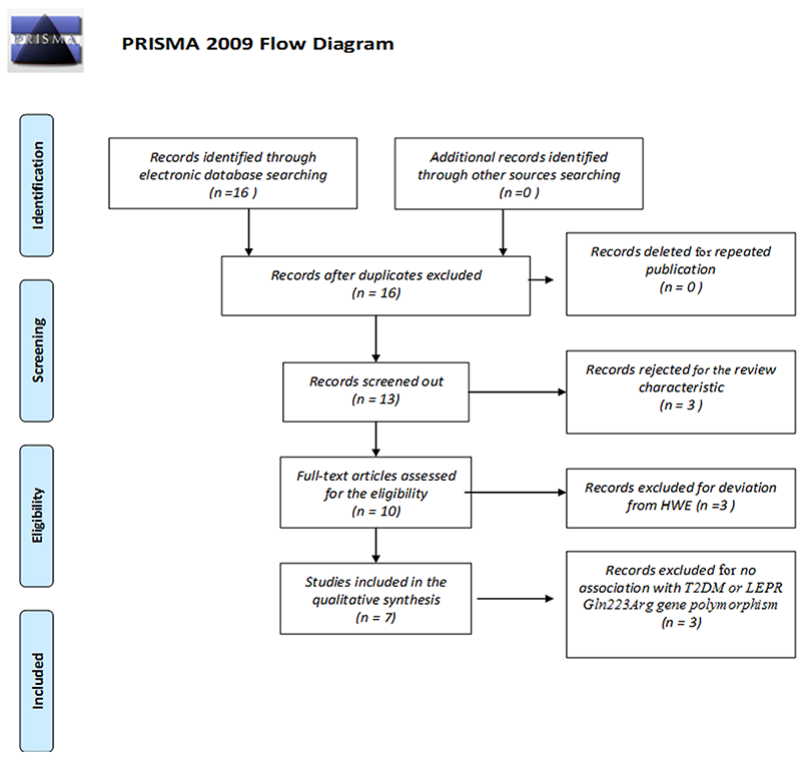
PRISMA 2009 flow diagram

Publication years of the included studies ranged from 2002 to 2014 (last research updated on May 15, 2017). Of the 16 papers retrieved by the initial search, seven met inclusion criteria. Among the nine excluded studies, three studies were excluded due to control groups that deviated from Hardy-Weinberg equilibrium (HWE). Three studies were reviews and an additional three studies did not analyze either T2DM or the *LEPR* Gln223Arg gene polymorphism. Among the ten included studies, three studies deviated from the HWE [8–10] (Figure 1). The full data set was represented by 2,084 T2DM patients and 1,283 controls (Table 1) [5–7, 11–14] from seven different provinces: Beijing, Jiangsu, Yunnan, Hubei, Heilongjiang, Jilin and Taiwan. All of the included studies received at least seven stars using NOS.

**Table 1:** Characteristics of the investigated studies of the association between *leptin receptor* (*LEPR)* gene Gln223Arg polymorphism and type 2 diabetes mellitus (T2DM) in the Chinese population

Author	Year	Region	T2DM			Control			Matching criteria	sample size(T2DM /control)	P_HWE_
			GG	GA	AA	GG	GA	AA			
Liao WL [11]	2012	Taiwan	796	194	8	36	8	1	Ethnicity	998/45	>0.05
Fang MZ [6]	2011	Yunnan	169	64	6	150	116	20	Ethnicity, TC, LDL	239/286	>0.05
Shi XH [7]	2005	Beijing	283	49	1	310	80	5	Ethnicity	333/395	>0.05
Xie P [12]	2002	Jiangsu	74	10	2	69	17	0	Ethnicity	86/86	>0.05
Ying J [5]	2009	Hubei	80	24	0	89	21	1	Ethnicity	104/111	>0.05
Gan RT [13]	2012	Heilongjiang	111	33	4	121	47	4	Age, sex, ethnicity	148/172	>0.05
Jiang B [14]	2014	Jilin	140	34	3	117	33	3	Age, sex, HDL, LDL, ethnicity	177/153	>0.05

T2DM: type 2 diabetes mellitus; TC: total cholesterol; LDL: low density lipoprotein-cholesterol; HDL: high density lipoprotein-cholesterol;

PCR-RFLP: polymerase chain reaction-restriction fragment length polymorphism;

PCR-RFLP and case-control study design were adopted in the above studies.

### Pooled analyses

**Figure 2:**
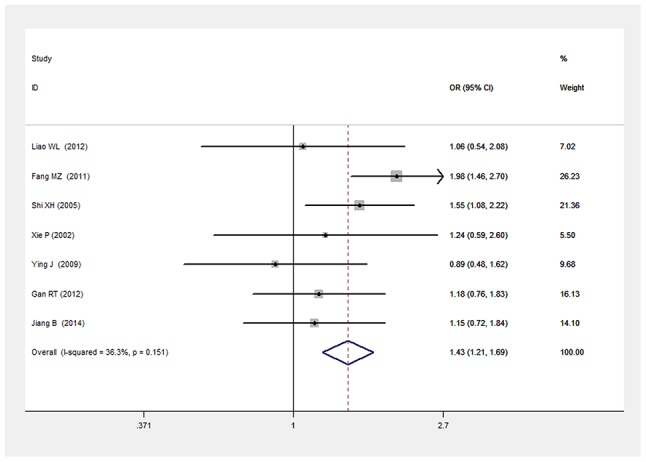
Forest plot of T2DM associated with *LEPR* Gln223Arg gene polymorphism under an allele genetic model (G vs. A)

**Figure 3:**
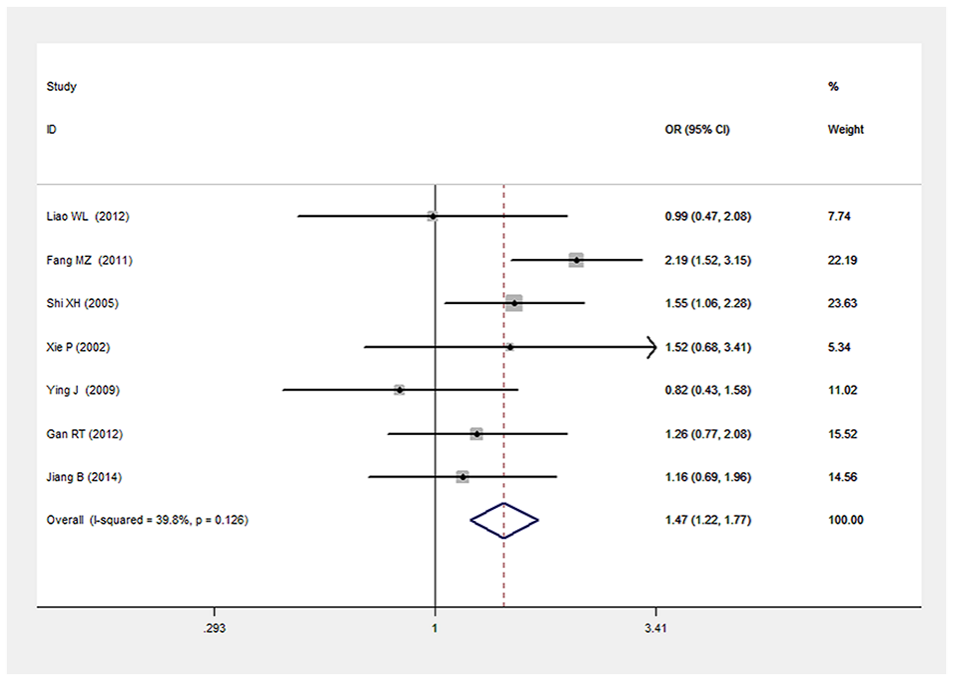
Forest plot of T2DM associated with *LEPR* Gln223Arg gene polymorphism under a dominant genetic model (GG vs. GA+AA)

**Figure 4:**
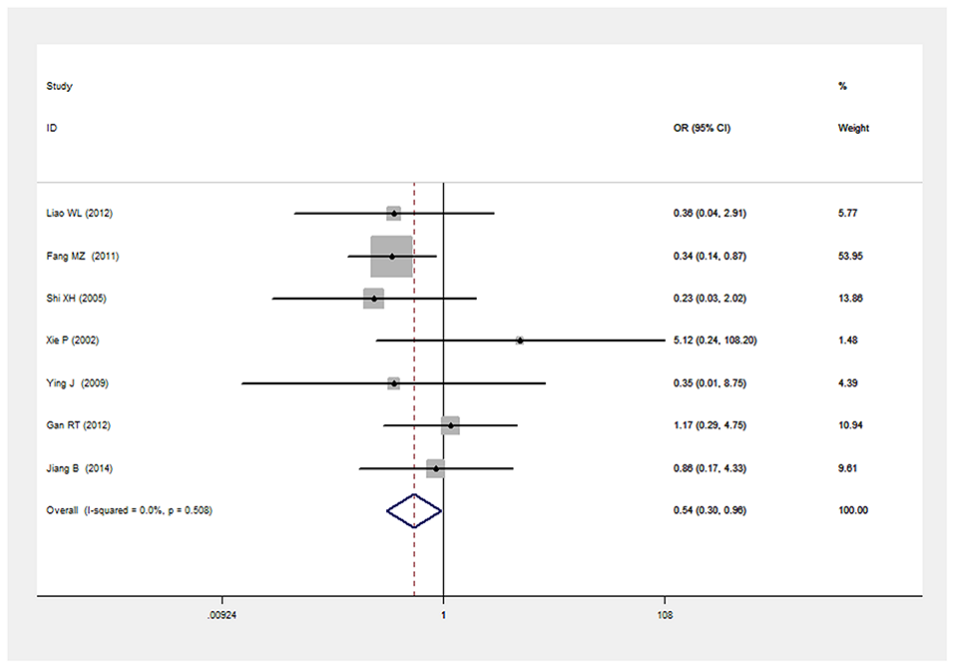
Forest plot of T2DM associated with *LEPR* Gln223Arg gene polymorphism under a recessive genetic model (AA vs. GG+GA)

**Figure 5:**
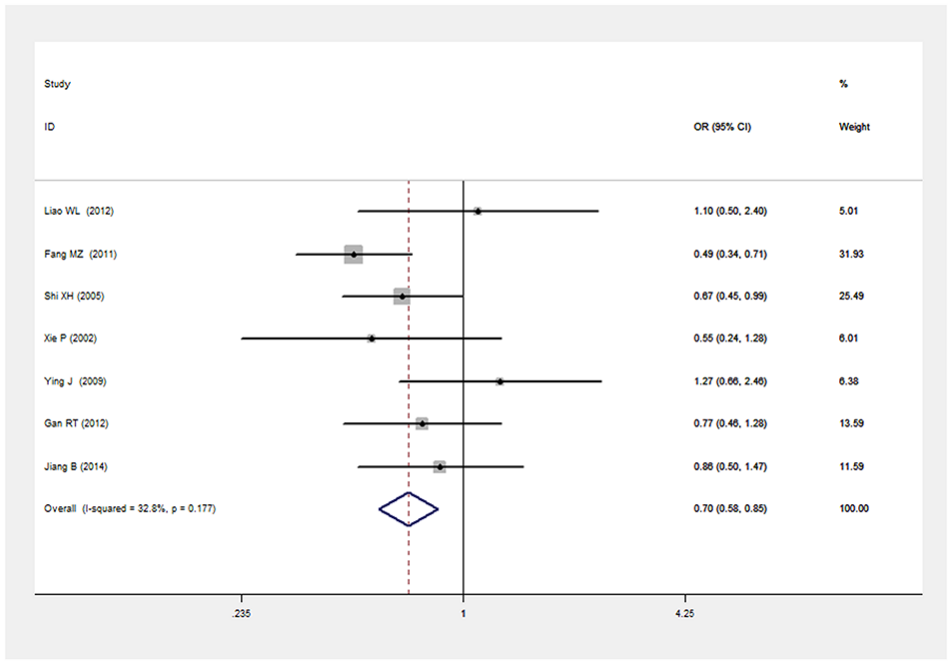
Forest plot of T2DM associated with *LEPR* Gln223Arg gene polymorphism under a heterozygous genetic model (GA vs. GG)

**Figure 6:**
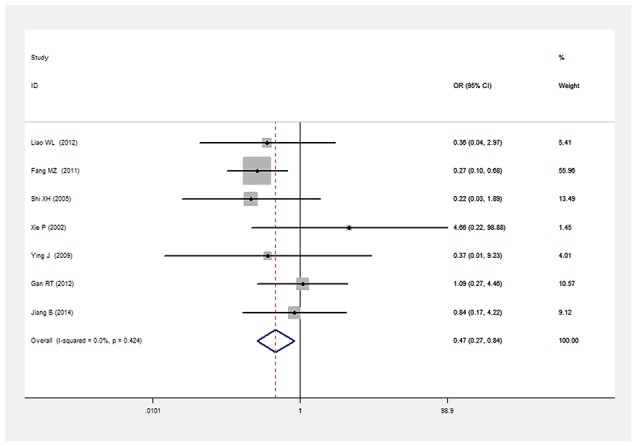
Forest plot of T2DM associated with *LEPR* Gln223Arg gene polymorphism under a homozygous genetic model (AA vs. GG)

There was a significant relationship between *LEPR* Gln223Arg gene polymorphism and T2DM in the Chinese population under allele (OR: 1.432, 95% CI: 1.211-1.694, P=2.67×10^-5^), dominant (OR: 1.466, 95% CI: 1.215-1.769, P=6.33×10^-5^), recessive (OR: 0.539, 95% CI: 0.303-0.960, P=0.036), heterozygous (OR: 0.700, 95% CI: 0.577-0.849, P=3.06×10^-4^), homozygous (OR: 0.472, 95% CI: 0.265-0.839, P=0.011). No significant heterogeneity was detected under all five genetic models (P>0.05) (Figures 2–6, Table 2).

**Table 2:** Summary of meta-analysis of association between *LEPR* gene Gln223Arg polymorphism and T2DM in the Chinese population

Genetic model	Pooled OR (95% CI)	Z value	P value	Study number	T2DM size	control size	*P*_heterogeneity(*I2%*)_
Allele genetic model	1.432(1.211-1.694)	4.20	2.67×10^-5^*	7	2084	1283	0.151(36.3%)
Dominant genetic model	1.466(1.215-1.769)	4.00	6.33×10^-5^*	7	2084	1283	0.126(39.8%)
Recessive genetic model	0.539(0.303-0.960)	2.10	0.036*	7	2084	1283	0.508(0%)
Heterozygous genetic model	0.700(0.577-0.849)	3.61	3.06×10^-4^*	7	2084	1283	0.177(32.8%)
Homozygous genetic model	0.472(0.265-0.839)	2.56	0.011*	7	2084	1283	0.424(0%)

*P<0.05.

CI: confidence interval; OR: odds ratio; T2DM size: the total number of T2DM cases; control size: the total number of control group; allele genetic model: G vs. A; dominant genetic model: GG vs. GA+AA; recessive genetic model: AA vs. GA+ GG; heterozygous genetic model: GA vs. GG; homozygous genetic model: AA vs. GG.

### Bias diagnostics

**Figure 7:**
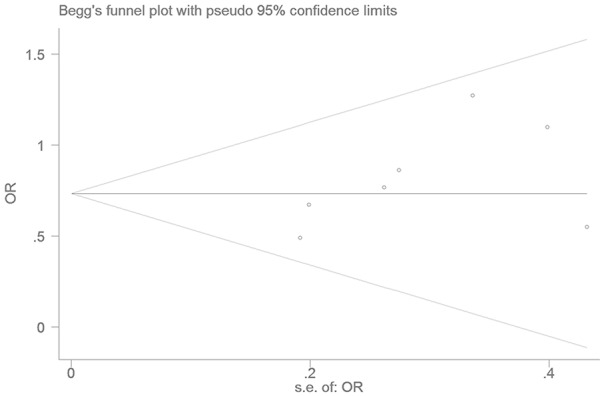
Funnel plot for studies of the association of T2DM associated and *LEPR* Gln223Arg gene polymorphism under a heterozygous genetic model (GA vs. GG) The horizontal and vertical axis correspond to the OR and confidence limits. OR: odds ratio; SE: standard error.

The publication bias of the individual studies was assessed by using an Egger’s test. No significant difference was detected, indicating that there was minimal publication bias in the current meta-analysis under the heterozygous genetic model (T = 1.77, P= 0.136) (Figure 7).

## DISCUSSION

In the current meta-analysis, we observed a significant association between the *LEPR* Gln223Arg gene polymorphism and T2DM in the Chinese population under the allele (OR: 1.432), dominant (OR: 1.466), recessive (OR: 0.539), heterozygous (OR: 0.700), and homozygous (OR: 0.472) genetic models. The fixed genetic model was used exclusively due to the lack of heterogeneity between studies. We, therefore, concluded that the Chinese individuals with the G allele of *LEPR* Gln223Arg gene polymorphism may be predisposed to T2DM.

T2DM is a polygenic disease which is closely related with obesity, hypertension, gout, and lipid metabolism disorder. T2DM is clinically called insulin resistance (IR) syndrome, the core of which is the IR. Recently many studies have shown that the leptin resistance might still exist in the IR patients at the same time [15]. Leptin is a fat-derived hormone which could play a role in the regulating the energy metabolism and body lipid homeostasis after it is combined with the LEPR located in the hypothalamus and adipose tissue. Hence, the *LEPR* gene is also called T2DM gene.

What mechanisms can explain the increased risk of T2DM associated with this polymorphism? One possible mechanism is that the glutamine residue may cause reduced LEPR expression on the plasma membrane impairing signal transduction through the receptor. It also could increase risk of T2DM by influencing neurological function of the vagus nerve.

The vagus nerve sensibility for the insulin secretion were reduced and further led to the IR, disrupting, not only glucose metabolism, but also fat metabolism. The IR not only makes the glucose metabolism disturbance, but also the fat metabolism disturbance. Although these mechanisms have yet to be demonstrated physiological, they provide a plausible rationale for the results from this study [5].

Liu et al and Su et al. both performed meta-analysis on the association between *LEPR* Gln223Arg gene polymorphism and T2DM in 2015 and 2016, respectively [16, 17]. They both concluded that *LEPR* Gln223Arg gene polymorphism had no effect on the susceptibility with T2DM. Both of these papers, however, do not take into account differences in ethnicity. Liu’s meta-analysis grouped all Asians, including Koreans, Indians, Malaysia, and Chinese in to a single subgroup while Su’s meta-analysis analyzed data that combined Asians and Europeans. These meta-analyses also included studies with the controls’ genotype number that deviated from HWE, such as individual studies by Zhang, Zhao, and Liu [8–10]. These factors combined may help account for the differences in results and also lend further credibility to the results of this study.

Our study is not without limitations, however, as our results do not replace the need for more large-scale studies on the association of T2DM with *LEPR* Gln223Arg gene polymorphism. It also fails to take into account other possibly significant factors that play a role in determining plasma LEPR levels [18].

In conclusion, we found a significant association between the *LEPR* Gln223Arg gene polymorphism and increased T2DM risk in the Chinese population. Chinese carriers of the G allele of *LEPR* Gln223Arg gene polymorphism may be more susceptible to T2DM. This conclusion clarifies the mixed results on the topic and develops our understanding of T2DM so that in the future, we can develop personalized treatment strategies for our patients. Taking account the above mentioned limitations, more research is required to verify this conclusion.

## MATERIALS AND METHODS

### Publication search and inclusion criteria

The keywords LEPR, Q223R or Gln223Arg, rs1137101, T2DM, diabetic, and diabetes were searched in on PubMed, the Web of Science, Embase, the China National Knowledge Infrastructure, the China Biological Medicine Database, the VIP database, and the Wanfang database.

In order to meet inclusion criteria, the paper had to a) evaluate the association between *LEPR* Gln223Arg gene polymorphism and T2DM b) diagnose T2DM based on criteria determined by the World Health Organization in 1999 (i.e. definite medical history of T2DM medical history and fasting plasma glucose > 7.0mmol/L or plasma glucose > 11.1mmol/L two hours after oral glucose tolerance test) c) be case-controls or cohort studies published in official journals d) have a control group whose genotype is at HWE. e) have cases where allele and genotype frequencies and their corresponding controls were available.

Studies that evaluated the association of *LEPR* gene Gln223Arg polymorphism with other related conditions, such as Type 1 diabetes mellitus, obesity, or hypertension, were excluded. Participants with diseases of the heart, liver, kidney, and the urinary tract were also excluded. The Newcastle-Ottawa Scale (NOS) was used to assess the quality of the included studies.

### Data extraction

Included data was abstracted through a standardized protocol of three researchers. Two researchers identified studies for inclusion independently, while a third acted as a mediator to resolve any disagreements between the two. Studies that violated the inclusion criteria, were published repeatedly, or lacked adequate data were rejected. Data sets used by the same authors for multiple papers were included in our analysis once. Table 1 displays the first author’s name, publication year, region, matching criteria, genotype number, and total number of cases and controls.

### Statistical analysis

Statistical analysis was performed using STATA 12.0 software (StataCorp, College Station, TX). Five genetic models, allelic (total G vs. total A), dominant (GG vs. GA+AA), recessive (AA vs. GA+GG), homozygous (AA vs. GG), and heterozygous genetic models (GA vs. GG) were adopted for the current meta-analysis. The association of *LEPR* Gln223Arg gene polymorphism with T2DM was evaluated using odds ratio (OR) and their corresponding 95% confidence intervals. Heterogeneity between the individual studies was calculated by the Chi-square-based Q-test [19]. Due to the lack of heterogeneity among the studies, the pooled OR was estimated using the fixed-effects model (the Mantel-Haenszel method) [20]. Z-test was used to determine the pooled OR and the significance was set at P<0.05 level. The Fisher′s exact test was used to evaluate the HWE. The potential publication bias was assessed by using the funnel plot. The funnel plot asymmetry was evaluated by using the Egger’s linear regression test on the OR. The significance was set at P<0.05 level in all of the above mentioned tests [21].

## Abbreviations

LEPR: leptin receptor
T2DM: Type 2 diabetes mellitus
ORs: odds ratios
95% CI: 95% confidence intervals
DM: diabetes mellitus
NPY: neuropeptide Y
HWE: Hardy-Weinberg equilibrium
IR: insulin resistance
NOS: Newcastle-Ottawa Scale
